# Environmental stresses inhibit splicing in the aquatic fungus *Blastocladiella emersonii*

**DOI:** 10.1186/1471-2180-9-231

**Published:** 2009-10-29

**Authors:** Raphaela Castro Georg, Rosane MP Stefani, Suely Lopes Gomes

**Affiliations:** 1 Departamento de Bioquímica, Instituto de Química, Universidade de São Paulo, Brazil; 2 Departamento de Bioquímica e Biologia Molecular, Instituto de Ciências Biológicas, Universidade Federal de Goiás, Brazil

## Abstract

**Background:**

Exposure of cells to environmental stress conditions can lead to the interruption of several intracellular processes, in particular those performed by macromolecular complexes such as the spliceosome.

**Results:**

During nucleotide sequencing of cDNA libraries constructed using RNA isolated from *B. emersonii *cells submitted to heat shock and cadmium stress, a large number of ESTs with retained introns was observed. Among the 6,350 ESTs obtained through sequencing of stress cDNA libraries, 181 ESTs presented putative introns (2.9%), while sequencing of cDNA libraries from unstressed *B. emersonii *cells revealed only 0.2% of ESTs containing introns. These data indicate an enrichment of ESTs with introns in *B. emersonii *stress cDNA libraries. Among the 85 genes corresponding to the ESTs that retained introns, 19 showed more than one intron and three showed three introns, with intron length ranging from 55 to 333 nucleotides. Canonical splicing junctions were observed in most of these introns, junction sequences being very similar to those found in introns from genes previously characterized in *B. emersonii*, suggesting that inhibition of splicing during stress is apparently a random process. Confirming our observations, analyses of *gpx3 *and *hsp70 *mRNAs by Northern blot and S1 protection assays revealed a strong inhibition of intron splicing in cells submitted to cadmium stress.

**Conclusion:**

In conclusion, data indicate that environmental stresses, particularly cadmium treatment, inhibit intron processing in *B. emersonii*, revealing a new adaptive response to cellular exposure to this heavy metal.

## Background

Exposure to environmental stresses leads to the disruption of many intracellular processes, in particular those carried out by macromolecular complexes, which are extremely sensitive to perturbation by stress conditions [1]. An example of a macromolecular complex that could be affected by environmental stresses is the spliceosome, which is responsible for intron excision, an important cellular process. The spliceosome is a multicomponent complex formed by hundreds of proteins and five small nuclear RNAs (U1, U2, U4, U5 and U6 snRNAs) assembled on the newly synthesized precursor messenger RNA (pre-mRNA) [2,3]. This complex catalyzes a two-step transesterification reaction required to remove the introns and connect the exons [4,5]. Intron splicing is a precisely regulated process, where only four intron sequences guide spliceosome machinery. They are: the exon-intron junction at the 5' and 3' end of the introns (5'ss - GT, 3'ss - AG); the branch site sequence located upstream of the 3'ss; and the polypyrimidine tract located between the 3'ss and the branch site [6].

The aquatic fungus *Blastocladiella emersonii *belongs to the Chytridiomycete class, which is at the base of the fungal phylogenetic tree [7,8]. Throughout its life cycle this fungus suffers dramatic biochemical and morphological changes, especially during two distinct stages of cell differentiation: germination and sporulation [9]. Both stages can be induced with a high degree of synchrony, and drastic changes in the patterns of RNA and protein syntheses are observed throughout the fungus life cycle. In nature, *B. emersonii *can be exposed to distinct environmental conditions, as temperature fluctuations and presence of heavy metals, as cadmium, that could lead to the disruption of some cellular functions.

It was previously shown that the splicing machinery is sensitive to thermal stress, as exposure of *Saccharomyces cerevisiae *cells to heat shock at 42°C leads to the accumulation of pre-mRNA species containing unspliced introns [10]. This splicing inhibition was also observed in a variety of species from yeast to humans, including *B. emersonii *[10-14]. However, the splicing machinery seems to be more thermoresistant in *B. emersonii *because at the lethal temperature of 42°C, when cell viability falls to less than 1% and protein synthesis is decreased by more than 95% [15], splicing is only partially inhibited in this fungus (30% inhibition) [13]. In yeast and *Drosophila melanogaster *at extreme temperatures splicing is inhibited more than 70% [10,11].

Although the effects of heat shock in the splicing machinery have been known for more than two decades [11], there is little information in the literature about how cadmium affects this complex. Cadmium (Cd^2+^) is a divalent cation present in polluted environments, which causes oxidative stress, lipid peroxidation and mutagenesis in the cells [16,17]. However, the molecular mechanisms by which cadmium leads to reactive oxygen species production and oxidative stress are largely unknown and are probably indirect. The mechanism usually proposed for cadmium toxicity is its binding to cellular proteins, resulting in the inhibition of some essential enzymes. As cadmium has high affinity for thiol groups, it is thought to bind accessible cysteine residues in proteins [16]. Another possible effect of cadmium exposure is the displacement of zinc and calcium from metalloproteins, leading to inhibition of these important proteins [16-18]. In this way, the presence of cadmium in the cells could affect, in theory, any biological process including the spliceosome machinery.

In this study, we demonstrate that environmental stresses, in particular, cadmium treatment inhibits splicing in the aquatic fungus *B. emersonii*. This inhibition is dose-dependent since we observed more unspliced mRNAs when higher cadmium concentrations were used. Thus, this work shows a new deleterious effect in RNA processing machinery when cells are exposed to cadmium.

## Methods

### Construction of cDNA libraries from stressed cells

ESTs analyzed in this work were obtained through the sequencing of three different cDNA libraries constructed from cells of *B. emersonii *submitted to heat shock and cadmium stress. The description of RNA extraction, cDNA library construction and EST sequencing is shown in [19]. Briefly, cDNA libraries were constructed from RNA samples isolated from sporulating cells exposed to heat shock at 38°C from 30 to 60 min after starvation (HSR library) or to 50 μM CdCl_2 _during the same period (CDM library) and from sporulating cells exposed to 100 μM CdCl_2 _from 60 to 90 min after starvation (CDC library).

### Identification of putative introns in *B. emersonii *ESTs

To identify putative introns, all ESTs obtained from the sequencing of the HSR, CDM and CDC cDNA libraries were grouped using Cap3 program [20]. The unigenes obtained (contigs plus singlets) (BeSAS - *B. emersonii *Stress Assembled Sequences) were compared with *B. emersonii *EST databank (BeAS - *B. emersonii *Assembled Sequences) using BlastN tool [21]. BeAS databank was generated from the sequencing of cDNA libraries constructed using RNA samples obtained from cells at different *B. emersonii *life cycle stages and that were not submitted to stress conditions [22,23]. BeSAS unigenes that presented extended regions of nucleotide identity with BeAS unigenes separated by regions that do not presented any nucleotide identity were pre-selected to be analyzed. We performed a search for canonical splicing junctions in these pre-selected BeSAS unigenes as well as for sequences corresponding to the putative branch site.

### Identification of putative genes encoding mRNA processing proteins in *B. emersonii*

We grouped all ESTs sequenced in *B. emersonii *transcriptome project (ESTs from stress and non-stress cDNA libraries) by using Cap3 program (BeSCAS - *B. emersonii *Stress and Cycle Assembled Sequences) and annotated the putative genes according to Gene Ontology (GO) terms. For more details, see references [19,23]. All BeSCAS genes that were annotated to the GO term "mRNA processing" (GO:0006397) were selected to be manually analyzed.

### Northern blot analysis

Total RNA was isolated from synchronized *B. emersonii cells *during sporulation, maintained at their physiological temperature (27°C) or exposed to heat shock (38°C during 30 min) and cadmium (50 μM CdCl_2 _and 100 μM CdCl_2 _during 30 min) using TRIzol reagent (Invitrogen) according to manufacturer's instructions. Gel electrophoresis and blotting were performed as described in [24].

### S1 protection assays of hsp70-1 RNA

The 5' end-labeled probe was prepared by labeling an 18-nt primer with [γ-^32^P] ATP and T4 polynucleotide kinase, which was then annealed to a single-stranded DNA from M13mpl8 containing a 2.3 kb BamHl-Sacl fragment of *hsp70-1 *genomic clone (coding strand) [13], and extended using the PolIk. The extending reaction was carried out in 12.5 mM Tris-HCl buffer pH 8.0 containing 6.25 mM MgC1_2_/25 μM dATP, dCTP, dTTP and dGTP and 5 units of PolIk. After incubation for 15 min at room temperature the enzyme was inactivated at 65°C for 10 min. The probe (4 × 10^5 ^cpm) was ethanol precipitated with 50 μg of total RNA isolated from cells at different stages of the life cycle and from cells submitted to different concentrations of CdCl_2_. The pellet was then suspended in 28 μl of formamide and 7 μl of 40 mM Pipes buffer, pH 6.4, containing 400 mM NaC1/1 mM EDTA. After boiling the samples for 10 min, the annealing was carried out for 3 h at 52°C. The samples were then diluted with 350 μl of 30 mM Na-acetate buffer pH 4.6/250 mM NaCl/1 mM ZnSO_4_/20 μg per ml calf thymus DNA, and digested at 37°C for 30 min with 50 units of S1 nuclease (GE Healthcare). The nucleic acids were ethanol precipitated, suspended in 4 μl of formamide sample buffer, and analyzed in 7 M urea-6% PAGE followed by autoradiography. The fragments were sized by comparison with Mspl digest ^32^P-labeled pBR322 DNA.

## Results

### *B. emersonii *stress cDNA libraries are enriched in ESTs with introns

The sequencing of ESTs from cDNA libraries constructed from *B. emersonii *cells submitted to heat shock and cadmium stress suggested that introns have been retained in several of them. Therefore, we speculated that the stress conditions used to construct these libraries could be affecting mRNA splicing in *B. emersonii*. To test this hypothesis, we initially identified all the ESTs sequenced from stress cDNA libraries that contained putative introns (iESTs).

Among the 6,350 ESTs sequenced from the stress libraries, 181 ESTs (corresponding to 105 introns retained from 85 distinct genes - Additional file 1) presented putative introns (2.9%), while in the sequencing of cDNA libraries from cells not submitted to stresses it was verified that only 0.2% of the ESTs contained putative introns (Table 1). These data are consistent with our hypothesis and indicate that there is an enrichment of ESTs with introns in *B. emersonii *stress cDNA libraries. Interestingly, if we consider the cDNA libraries separately, we observe a more pronounced enrichment of iESTs (4.9%) in the cDNA library constructed from cells submitted to the higher concentration of cadmium (100 μM) (Table 1).

**Table 1:** Number of iESTs sequenced from stress cDNA libraries.

cDNA library	Total of ESTs with introns	Total of ESTs sequenced	Ratio (%)
HSR (Heat shock)	34	3,070	1.1
CDM (CdCl_2 _50 μM)	65	2,400	2.7
CDC (CdCl_2 _100 μM)	83	1,920	4.3
**Total (stress)**	**181**	**6,350**	**2.9**
**Total (normal)**	**45**	**23,350**	**0.2**

iESTs corresponds to ESTs with retained introns; normal corresponds to cDNA libraries from unstressed cells.

**Figure 1:**
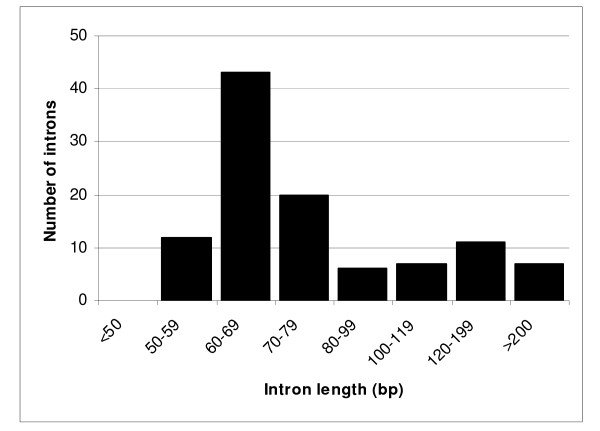
**Length distribution of 105 *B. emersonii *introns in ESTs from stress libraries**.

**Figure 2:**
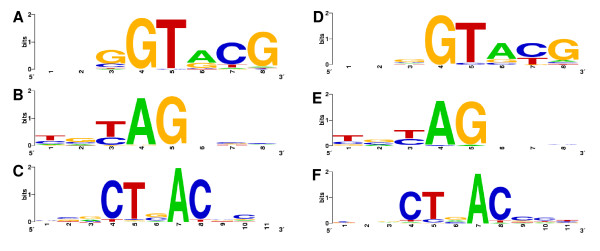
**Sequence conservation in *B. emersonii *introns**. Consensus sequences for (A) 5' exon-intron junctions, (B) 3' intron-exon junctions and (C) putative branch point sequences were calculated based on 105 introns from ESTs obtained through sequencing of stress cDNA libraries using WebLogo server http://weblogo.berkeley.edu. The consensus sequences for (D) 5' exon-intron junctions, (E) 3' intron-exon junctions and (F) putative branch point from ESTs obtained through sequencing of non-stress cDNA libraries are also shown. In this case, the consensus sequences were calculated based on 35 introns. The intron sequences start at position four in (A) and (D), and end at position 5 in (B) and (E).

Among the 85 genes corresponding to the ESTs that retained introns, 19 showed more than one intron and three showed three introns. The intron length ranged from 55 to 333 nucleotides (Figure 1), most of the introns being between 60-79 nt long. To further characterize these putative introns we performed a search for the canonical splicing sites in the regions adjacent to intron sequences and also for the conserved sequence of the putative branch site, which is involved in lariat formation and intron splicing [25]. We detected the conserved dinucleotides at each end of the introns (GT at the 5' end and AG at the 3' end) in 102 of the 105 putative introns (Figure 2A, Additional file 1). All introns analyzed also presented a sequence similar to the conserved sequence (CTAAC) of the branch site. We performed the same search for the putative introns detected in ESTs from non-stress cDNA libraries and the result was very similar (Figure 2B). In addition, all nine previously characterized genes of *B. emersonii *containing introns showed the canonical splicing sites and a conserved branch site sequence [13,26-33].

These data show that canonical splicing junctions observed in most of the iESTs obtained through the sequencing of stress libraries are not different from other splicing junctions present in introns of genes previously characterized in *B. emersonii*, and also not different from introns retained in ESTs from non-stress libraries. This suggests that the mRNAs that had their splicing inhibited by stress were probably randomly affected or at least if there is a selection for some mRNAs, it is not based in differences in their splicing sites.

If we consider that selective inhibition of splicing could be a post-transcriptional regulatory mechanism to respond to stressful conditions, we would expect that a group of genes should have their mRNA processing inhibited to enhance the mRNA processing of other genes that could be more important for the response of *B. emersonii *to stress. However, when we analyzed the genes corresponding to the ESTs with introns retained, we did not observe a pattern among them (Additional file 1). On the contrary, some important genes as those encoding HSPs (Heat Shock Proteins), Glutathione-S-transferases and Thioredoxins that are induced in response to stress conditions [19], also presented iESTs sequenced from stress cDNA libraries.

Further evidence that is consistent with this idea is the fact that for 30% of the iESTs, at least one EST sequenced from stress libraries corresponding to the same gene did not retain the intronic sequences, i.e., the corresponding mRNA was correctly processed (Additional file 1).

### The spliceosome genes are not repressed under heat shock and cadmium stress

The inhibition of mRNA splicing caused by heat shock and cadmium treatment could be due to a decrease in the expression of genes encoding proteins of the spliceosome complex, leading to a reduction in the levels of the proteins forming the spliceosome. To test this hypothesis we identified all genes coding for spliceosome proteins that were present in *B. emersonii *EST database [19,22,23]. We observed 41 distinct genes (corresponding to 91 ESTs) encoding proteins involved in mRNA processing in this fungus (Additional file 2). To verify if these genes were up- or down-regulated during stress, we used the expression profile data of microarray assays of *B. emersonii *cells submitted to cadmium and heat shock, previously published by our group [19]. Among the 41 genes of *B. emersonii *related to mRNA processing, 29 were present on the microarray slide and only two of them were shown to be differentially expressed in response to cadmium or heat shock. One was induced by heat shock (BeE60H22E01 - snRNP core protein SMX5d) and the other (BeE60N15H01 - putative small nuclear ribonucleoprotein Sm-D1) was repressed by cadmium treatment [19,23].

The 41 genes observed through our search certainly do not correspond to all genes involved in mRNA processing in *B. emersonii*, since it has been shown that the spliceosome machinery is formed by hundreds of proteins in eukaryotes [2]. However, we believe that our set of genes is a significant part of those that encode proteins of the mRNA processing complex in *B. emersonii*. Nevertheless, we observed that only one gene was repressed under stress conditions. Thus, our data suggest that inhibition of mRNA splicing after cadmium and heat stress in this fungus is not due to a global repression of the genes involved in the splicing process under these conditions.

One of the possible effects of cadmium that lead to toxicity in cells is its capacity of displace zinc (Zn^2+^) and calcium (Ca^2+^) from proteins that need these cations to perform their functions [16,34,35]. So, the inhibition of splicing by cadmium in *B. emersonii *could be due to the substitution of zinc in proteins involved in mRNA processing, which could lead to impairment or even to loss of their function. Considering this hypothesis, we evaluated if among *B. emersonii *spliceosome proteins there were some that possessed zinc-binding domains, as zinc finger or zinc-related motifs, which could be affected by the presence of cadmium inside the cells.

Comparing the deduced amino acid sequences from the 41 mRNA processing-related genes from *B. emersonii *with a protein family database (PFAM) [36], we observed two proteins with putative zinc-related domains. They encode the cleavage and polyadenylation specificity factor 5 (BeCSAS2344) and the pre-mRNA splicing factor Cwc2 (BeE30N19E11) [22]. The former protein has a THAP domain, a putative DNA-binding domain that probably also binds a zinc ion, and the second protein has a zinc-finger domain. The presence of proteins that possess zinc-related domains has also been reported in the spliceosome of other organisms [37-40], indicating that this type of protein is a common component of the splicing machinery and could be the target of zinc displacement by cadmium.

### Splicing of hsp70-1 intron is inhibited by cadmium treatment but not by hydrogen peroxide

Previous studies showed that the processing of *B. emersonii hsp70-1 *intron is partially inhibited (30%) after heat treatment of the cells at the lethal temperature of 42°C [13]. The *hsp70-1 *gene was one of the genes that presented an iEST sequenced from libraries from cells exposed to cadmium stress (Additional file 1). However, we detected no *hsp70-1 *iEST in the heat shock cDNA library (HSR). This is probably due to the fact that in the construction of the heat shock cDNA library fungal cells were incubated at 38°C instead of the restrictive temperature of 42°C. To confirm the inhibition of *B. emersonii hsp70-1 *intron splicing by cadmium treatment, we performed S1 nuclease protection assays using a 5'end-labeled probe prepared as described in Materials and Methods. The probe was hybridized to total RNA isolated from cells submitted to cadmium treatment (250 μM). As a control of splicing inhibition, we also used total RNA isolated from cells submitted to heat shock at 38°C and 42°C.

**Figure 3:**
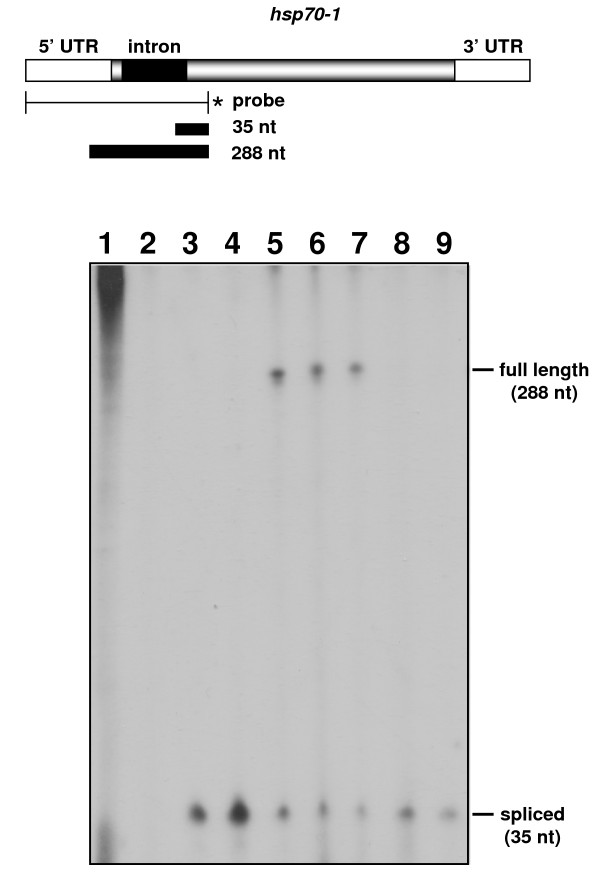
**Splicing of *hsp70 *mRNA is inhibited in *B. emersonii *cells exposed to cadmium**. Nuclease Sl protection assays were performed using the 5' end-labeled probe, depicted above the autoradiogram, and 50 μg of yeast tRNA in the presence or absence of nuclease S1 (lanes 1 and 2) as control, 50 μg of total RNA isolated from 60 min sporulating cells (lane 3), cells submitted to heat shock at 38°C and 42°C for 30 min (lanes 4 and 5, respectively), cells submitted to 250 μM CdCl_2 _for 30 min and 60 min (lanes 6 and 7, respectively), cells submitted to 500 μM H_2_O_2 _for 30 min and 60 min (lanes 8 and 9, respectively).

As depicted in Figure 3, a partial block in *hsp70-1 *intron splicing occurs after cadmium treatment suggesting that the presence of this heavy metal in cells impairs spliceosome function. The *hsp70-1 *intron was efficiently processed at 38°C but its splicing was partially inhibited when *B. emersonii *cells were incubated at 42°C, as previously shown by Stefani and Gomes [13] (Figure 3). To further test if the effect of cadmium on mRNA processing could be due to oxidative stress caused by the presence of the metal in the cells, we also analyzed the effect of hydrogen peroxide treatment on *B. emersonii hsp70-1 *intron splicing. We did not detect any inhibition of *hsp70-1 *intron processing when we performed the S1 nuclease protection assays using total RNA isolated from cells exposed to 0.5 mM hydrogen peroxide (Figure 3). These results suggest that splicing inhibition by cadmium treatment of *B. emersonii *cells is probably not due to oxidative stress caused by this heavy metal.

As S1 nuclease protection assays were performed using total RNA isolated from cells submitted to a higher concentration of cadmium (250 μM) than those used in the construction of the stress libraries (50 and 100 μM), we also performed these assays with RNA isolated from cells submitted to 25, 50 and 100 μM CdCl_2 _to verify the effect of different cadmium concentrations on *hsp70-1 *intron splicing. We observed a more pronounced block in the processing of *hsp70-1 *intron when *B. emersonii *cells were treated with 100 μM CdCl_2 _than with 50 μM CdCl_2_, while with 25 μM CdCl_2 _no inhibition of splicing was detected (Additional file 3). These results indicate that inhibition of splicing by cadmium treatment can be dose-dependent, consistent with our observation that a larger number of iESTs is found in the cDNA library constructed from cells submitted to 100 μM CdCl_2 _(CDC) than from cells exposed to 50 μM CdCl_2 _(CDM) (Additional file 1).

### Induction of thermotolerance by incubation at moderate temperatures restores splicing

**Figure 4:**
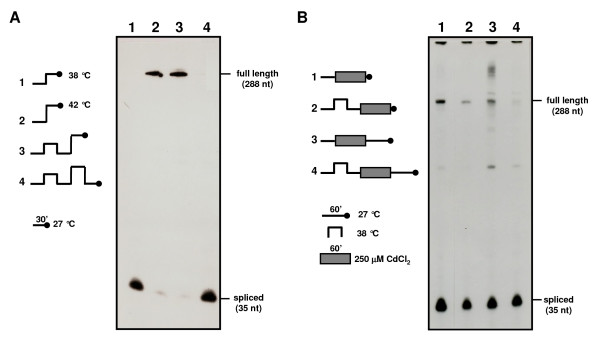
**Effect of thermotolerance on *hsp70 *mRNA splicing inhibition**. **A **-- Nuclease S1 protection assays were performed using a 5' end-labeled probe (the same used in Figure 3) and 50 μg of total RNA isolated from cells incubated at the following temperatures for 30 min: 27°C and 38°C (lane 1); 27°C and 42°C (lane 2); 27°C, 38°C, 27°C and 42°C (lane 3); 27°C, 38°C, 27°C, 42°C and 27°C (lane 4). **B **-- Cells incubated at 27°C for 30 min and then with 250 μM CdCl_2 _for 60 min (lane 1); cells incubated at 27°C for 30 min, at 38°C for 30 min, at 27°C for 30 min, and then with 250 μM CdCl_2 _for 60 min (lane 2); cells incubated at 27°C for 30 min, with 250 μM CdCl_2 _for 60 min and then at 27°C for 60 min (lane 3); cells incubated at 27°C for 30 min, at 38°C for 30 min, at 27°C for 30 min, with 250 μM CdCl_2 _for 60 min and then at 27°C for 60 min (lane 4).

To test whether a pretreatment at moderate heat shock temperatures could exert some effect on mRNA processing in *B. emersonii *cells, S1 nuclease protection assays were performed using RNA samples from cells incubated at 38°C for 30 min prior to exposure to extreme heat shock temperature (42°C) or cadmium treatment. In these experiments, it was possible to observe that splicing inhibition occurring at 42°C could be completely reversed if pre-incubation at 38°C was associated with incubation at 27°C for 30 min after exposure to the extreme heat shock temperature (Figure 4A), which could be considered a recovery period. Furthermore, protein synthesis was necessary during the entire experiment, as addition of cycloheximide (10 μg/ml) at any time during cell incubation at the various temperatures prevented complete recovery of the cells' capacity to carry out splicing of *hsp70-1 *intron (not shown). In particular, addition of cycloheximide before the pre-incubation step at 38°C, revealed that this treatment is essential for reversing splicing inhibition, as no spliced mRNA is detected under this condition (not shown). In the case of splicing inhibition due to exposure to cadmium, pre-incubation at 38°C prior to heavy metal treatment was also capable of reversing inhibition (Figure 4B), but complete recovery of the splicing capacity was observed only if exposure to cadmium was followed by incubation at 27°C in the absence of the metal (Figure 4B).

### Processing of gpx3 intron is inhibited by cadmium treatment

**Figure 5:**
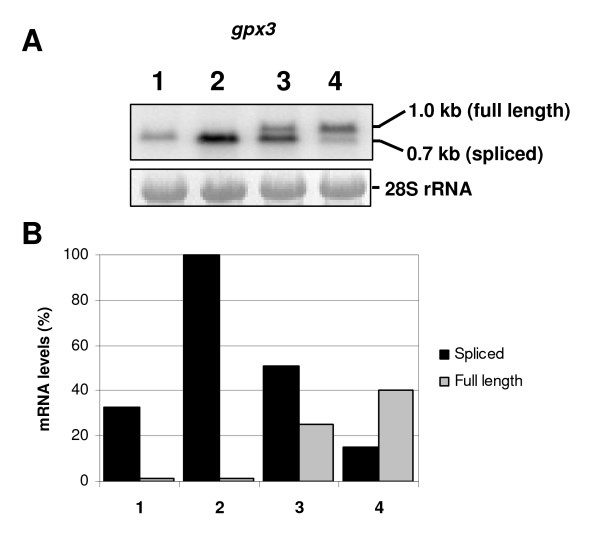
**Analysis of *gpx3 *mRNA in cells exposed to heat shock and cadmium stress**. **A**-Northern blot assay was performed using total RNA extracted from *B. emersonii *cells submitted to different cadmium concentrations or to heat shock. RNA extracted from cells 60 min after sporulation induction (lane 1). RNA extracted from cells submitted to heat shock (38°C) from 30 to 60 min (lane 2) after induction of sporulation. RNA extracted from cells 60 min after sporulation induction, incubated with 50 μM or 100 μM CdCl_2 _from 30 to 60 min (lanes 3 and 4, respectively) after sporulation induction. As a control of RNA levels, the 28S rRNA was shown. **B **-- Relative transcript levels of *gpx3 *mRNA determined by densitometry scanning of the autoradiogram shown in A. The figure legend (1, 2, 3 and 4) is the same depicted above.

To further verify the splicing inhibition by cadmium and its dose-dependent effect, we selected another gene to evaluate intron processing. The *gpx3 *gene encodes a Glutathione peroxidase and was chosen because its intron is 334-nt length, so unspliced mRNA could be easily differentiated from spliced mRNA in the Northern blot assays. The experiment was carried out using total RNA from *B. emersonii *cells submitted to heat shock (38°C), and cadmium (50 and 100 μM CdCl_2_). The unspliced form of *gpx3 *mRNA was detected only when cells were treated with cadmium, indicating a partial block in mRNA splicing (Figure 5). Inhibition of splicing was confirmed to be dose-dependent as a more pronounced inhibition was observed when *B. emersonii *cells were treated with the highest concentration of cadmium (100 μM). The unspliced form of *gpx3 *mRNA was not detected when cells were submitted to heat shock at 38°C, indicating that heat stress at this temperature produces no visible effect in *gpx3 *mRNA splicing. Interestingly, we observed that the *gpx3 *gene is induced both in response to cadmium and heat shock, an indication that this gene probably plays an important role in the response of *B. emersonii *to these two environmental stresses.

## Discussion

A constant challenge for unicellular organisms in nature is to maintain internal homeostasis despite abrupt and dramatic fluctuations in the external conditions. Sudden changes in the external environment can perturb the internal system of the cells, disrupting cellular functions. How organisms respond to these environmental changes to adapt to their surroundings and avoid cellular damages has been the subject of various research groups [19,41-44]. Nevertheless, most of those studies evaluated the effects of these environmental oscillations on gene expression, protein synthesis and cell phenotype [19,41-44], with only a few reporting the effects of stresses on the mechanism of pre-mRNA splicing [1,45].

This work describes for the first time, to the best of our knowledge, inhibition of splicing *in vivo *as an effect of cadmium treatment. The first evidence indicating this new effect of cadmium in *B. emersonii *cells was the observation of an enrichment of iESTs in the sequencing of the stress cDNA libraries. From 6,350 ESTs obtained through the sequencing of stress libraries, 2.9% correspond to iESTs, while in the sequencing of *B. emersonii *cDNA libraries, not submitted to environmental stresses, the percentage of iESTs was only 0.2%.

Two cDNA libraries were constructed from cells submitted to different cadmium concentrations and we observed that the higher the cadmium concentration the more iESTs were observed (4.3% of all ESTs sequenced from CDC library (100 μM CdCl_2_) corresponded to iESTs while in CDM library (50 μM CdCl_2_) this percentage was only 2.7%. Besides cadmium libraries, one cDNA library was constructed from cells submitted to heat shock in a moderate temperature (38°C) and even in this library we detected an enrichment of iESTs (1.1%). This observation is quite interesting since inhibition of splicing by thermal stress was already observed in *B. emersonii*, but only at lethal temperatures (42°C) [13]. These data indicate that intron splicing is affected in *B. emersonii *cells maintained at 38°C, but the effect observed in the splicing process is not so severe as the one detected in cells exposed to heat shock at 42°C [13] or cadmium treatment.

Sequencing of iESTs reported here provides considerable new information about *B. emersonii *intron structure and sequence, as only nine genes with their introns sequenced and deposited in GenBank database have been previously described in *B. emersonii *[13,26-33]. Thus, the present study contributes significantly to the knowledge about gene organization in this fungus.

Among the 85 genes whose corresponding mRNAs retained introns in the stress cDNA libraries, a total of 22% of them presented two or three introns. Fungal genes are commonly interrupted by few and small introns in comparison with metazoan genes. Intron density ranges from five to six per gene in basidiomycetes as *Cryptococcus neoformans *[46], from one to two per gene in recently sequenced ascomycetes as *Neurospora crassa *and *Magnaporthe grisea *[47,48], and less than 300 introns present in the entire *S. cerevisiae *genome [49], while human genes are interrupted in average by eight introns [50]. Thus, considering the number of introns reported here, *B. emersonii*'s gene structure appears to be more similar to that observed in ascomycetes. Further evidence suggesting that *B. emersonii *gene structure is more similar to ascomycetes is the average intron length observed in this aquatic fungus. We detected introns ranging from 55 to 333 nucleotides, an intron length more similar to that observed in the ascomycete species [49-51]. However, it is relevant to notice that even fungi belonging to the same class present different gene structures, as the case of *Ustilago maydis*, a basidiomycete that possesses an average number of introns per gene smaller than one [52,53].

To further characterize the intron structure of *B. emersonii *genes, we have identified the splicing junctions present in the introns sequenced from iESTs. We observed that most of the introns showed the canonical splicing sites and the consensus branch site sequence similar to those detected in introns from genes previously characterized in *B. emersonii*. These observations suggest that inhibition of splicing by stress in *B. emersonii *is probably a random process opposite to a selective inhibition of some specific pre-mRNAs based on different intron-recognition sequences.

The fact that *B. emersonii *possesses proteins involved in pre-mRNA processing containing zinc-related domains indicates that one possible mechanism by which cadmium inhibits splicing in this fungus could be the displacement of zinc ions from these proteins. This hypothesis is consistent with the fact that we did not observe a global repression in the transcription of genes encoding spliceosome proteins under these stress conditions. Additionally, the *hsp70-1 *gene intron was not found to be retained when *B. emersonii *cells were treated with hydrogen peroxide. These data suggest that splicing blockage is not due to an indirect effect of oxidative stress caused by cadmium. Furthermore, Shomron and collaborators [54] demonstrated that zinc is an essential factor for the second step of the splicing reaction, suggesting that putative zinc-dependent metalloproteins are required for this step of RNA splicing process. Interestingly, a recent report demonstrated that cadmium, a metal that presents many chemical similarities to zinc, in low quantities can restore *in vitro *mRNA splicing inhibited by zinc-depletion [55]. These results indicated that cadmium could effectively substitute zinc in metalloproteins, including those present in the spliceosome machinery [55]. Nevertheless, at higher concentrations the authors observed that cadmium caused the opposite effect, inhibiting splicing *in vitro *[55]. In fact, cadmium should exert a direct effect in the spliceosome complex, by displacing zinc, since at high concentrations of zinc, no splicing inhibition was observed, a higher cadmium concentration treatment being necessary to detect intron retention [55].

The mechanism of zinc displacement is not applicable to splicing inhibition by thermal stress. In this case, most probably inhibition is due to the unfolding of spliceosome proteins as a consequence of high temperature. Consistent with this hypothesis, it was observed that heat shock proteins (HSPs) are involved in the protection of the spliceosome complex at higher temperatures [56]. Yeast cells made thermotolerant by preincubation at 37°C completely protect spliceosome snRNPs complexes from disruption when subsequently exposed to a more severe stress at 42°C [56].

Interestingly, we also observed that in *B. emersonii *cells made thermotolerant by pretreatment at 38°C and later exposed to cadmium, mRNA processing is less affected than in cells not previously treated. One possible explanation of this thermoprotection effect in mRNA processing machinery is that during heat shock cells could be inducing the expression of proteins that are important to the response to temperature stress but that are also important in the response to cadmium treatment. In fact, during the response to heat shock, *B. emersonii *cells induce not only the expression of heat shock protein genes but also genes encoding several antioxidant proteins [19], which could be exerting a protective effect in cells subsequently exposed to cadmium. Indeed, we observed here that *B. emersonii gpx3 *gene, which encodes a Glutathione peroxidase, is highly induced in response to both heat shock and cadmium treatment.

Another possible explanation for splicing inhibition by cadmium and heat shock could be that under these conditions introns are retained in some genes just because they are alternatively spliced. However, this hypothesis does not hold as only 30% of the iESTs maintain their reading frames, and at least for the *hsp70-1 *gene the protein originated from this putative alternative splicing was not detected in western blots [13], indicating that the unspliced mRNA is not efficiently translated. It is important to notice that another process that could be affected by cadmium treatment resulting in intron retention is the machinery of nonsense-mediated decay, since this complex is responsible for the degradation of unspliced mRNAs in the cell [57].

In yeast, transcript-specific changes in splicing were observed in response to environmental stresses. For instance, it was shown that in response to amino acid starvation splicing of most ribosomal protein-encoding genes was inhibited, splicing being an important opportunity for regulation of gene expression in response to stress [45]. This kind of post-transcriptional regulation does not seem to be the case during splicing inhibition by heat and cadmium stresses in *B. emersonii*, as we did not observe a pattern among the genes whose pre-mRNA splicing was inhibited, indicating that there was no preference for transcripts that are involved in specific biological processes.

To confirm the inhibitory effect of cadmium in the spliceosome machinery we performed S1 nuclease protection and Northern blot assays to analyze the splicing of two different mRNAs (*hsp70-1 *and *gpx3*) under this stress condition. Inhibition of pre-mRNA splicing of both genes was observed when *B. emersonii *cells were submitted to cadmium, validating our sequencing data. Although intron retention could be a *B. emersonii *response to stress treatment, it is still unclear to us what kind of benefits this response could bring to the cell. In fact, the results do not seem to indicate that intron retention might be a regulatory mechanism under stress conditions. On the contrary, it is most probable that this event occurs randomly, being the most abundant mRNAs more affected, as those corresponding to genes induced in response to stresses.

## Conclusion

This work demonstrates that environmental stresses, mainly cadmium exposure, inhibit splicing in *B. emersonii*. The cellular effects of cadmium, which lead to its toxicity, have been investigated in recent years. These effects include generation of oxidative stress, lipid peroxidation, mutagenesis and others. However, until now no description of an effect of cadmium on the spliceosome machinery was reported. Thus, this study contributes to the elucidation of a new mechanism promoting cadmium toxicity to the cells.

## Authors' contributions

RCG carried out the construction and analysis of stress cDNA libraries, bioinformatics analysis, Northern blot experiments and drafted the manuscript. RMPS carried out S1 protection assays. SLG participated in study design and coordination and helped to draft the manuscript. All authors read and approved the final manuscript.

## Supplementary Material

Additional file 1 ***B. emersonii *genes corresponding to iESTs sequenced from stress cDNA libraries**. The table shows the ESTs sequenced that retained introns. Click here for file

Additional file 2 **Genes encoding spliceosome proteins in *B. emersonii*, annotated in GO category "mRNA processing"**. The table shows ESTs that participate in mRNA processing in *B. emersonii*. Click here for file

Additional file 3 **S1 protection assays of *hsp70 *mRNA in different cadmium concentrations**. The figure shows Sl protection assays of *hsp70 *mRNA using total RNA extracted from *B. emersonii *cells submitted to different cadmium concentrations. Click here for file
