# Supplementary material for: Environmental stresses inhibit splicing in the aquatic fungus Blastocladiella emersonii
Source: BMC Microbiol. 2009 Oct 29;9:231. doi: 10.1186/1471-2180-9-231 (PMC2773782; doi:10.1186/1471-2180-9-231)
Supplement: Additional file 1 — B. emersonii genes corresponding to iESTs sequenced from stress cDNA libraries. The table shows the ESTs sequenced that retained introns. [file 1471-2180-9-231-S1.pdf]

Supplemental Table 1 – *B. emersonii* genes corresponding to iESTs sequenced from stress cDNA libraries.

Gene ID	Annotation	5' SS	Branch site	3' SS	# of iESTs			Length (bp)	Biological process (GO)	# of ESTs (no intron)
					CDM	CDC	HSR			
BeSAS691*	6-pyruvoyltetrahydropterin synthase	G 'gtatg	cctcac	gacag' G	-	5	-	132	amino acid metabolism	
BeSAS582	Hypothetical protein	G 'gtacg	tctgac	cgcag' C	-	2	-	205	carbohydrate biosynthesis	
BeE90D08B09	Chitinase	A 'gtacg	actgac	tgtag' C	-	1	-	70	carbohydrate metabolism	15
BeE90D04F01	Septin 2	C 'gtacg	gctgac	cgcag' A	-	1	-	71	cell cycle	2
BeE60C05G05	Beta tubulin cofactor A	T 'gtgcg	gctcac	cgtag' T	1	-	-	58	chaperonin-mediated tubulin folding	
BeE60C12H07	Prenylcysteine carboxylmethyltransferase	T 'gtccg	gctgac	tctag' C	1	-	-	79	C-terminal protein amino acid methylation	
BeSAS333*	Dynein light chain	G 'gtgcg	actcat	tgtag' A	2	1	-	63	dendrite morphogenesis	
BeSAS327(1)*	Thioredoxin II	C 'gtacg	gctaac	accag' G	4	3	-	168	electron transport	8
BeSAS327(2)	Thioredoxin II	C 'gtacg	actcac	accag' G	4	3	-	70	electron transport	8
BeE90D04B07*	Thioredoxin domain	G 'gtgcg	gctgac	tatag' C	-	1	-	57	electron transport	
BeE60C34B08	Hypothetical protein	G 'gtact	actgac	tgtag' G	1	-	-	79	ER-associated protein catabolism	5
BeE90D12B01(1)	Glycolipid transfer protein	G 'gtccg	actgac	tgtag' A	-	1	-	62	glycolipid transport	2
BeE90D12B01(2)	Glycolipid transfer protein	G 'gtacg	gctgac	tgtag' C	-	1	-	64	glycolipid transport	2
BeE60H21C11*	Rabl2a protein	A 'gtacg	gctgac	cgtag' C	-	-	1	75	intracellular protein transport	
BeSAS509	Ferritin	G 'gtgcg	gctaac	tacag' G	2	2	-	71	iron ion homeostasis	32
BeSAS590(1)	Dynein light chain 2	C 'gtacg	gctcac	tgtag' A	-	2	-	76	microtubule-based process	11
BeSAS590(2)	Dynein light chain 2	G 'gtacg	actgac	ctgag' C	-	2	-	200	microtubule-based process	11
BeSAS605	Methyltransferase	C 'gtacg	gctgac	tgtag' C	-	2	-	64	phospholipid metabolism	3
BeE60C07D01*	Hypothetical protein	G 'gtacc	gctaac	catag' G	1	-	-	63	phospholipid metabolism	
BeE60C23G07	Tyrosine phosphatase	T 'gtatg	cctcac	cgtag' G	1	-	-	61	protein amino acid dephosphorylation	
BeSAS395*	50S ribosomal protein L28	C 'gtgag	actgac	cctag' T	1	-	2	57	protein biosynthesis	
BeSAS407*	Hsp70-1	G 'gtacg	gctcac	tctag' A	1	1	-	165	protein folding	12
BeSAS726*	Hsp60	G 'gttcg	actaat	tgtag' C	-	2	-	69	protein folding	
BeSAS568*	Hsp30	G 'gtacg	actcac	cgtag' A	2	1	-	69	protein folding	20
BeSAS762*	Hsp17	A 'gtacg	actcac	tgcag' A	1	3	-	96	protein folding	167
BeE90D15G01(1)	Hsp16	G 'gtgcg	actgac	tgcag' G	-	1	-	65	protein folding	8
BeE90D15G01(2)	Hsp16	G 'gtatg	cctaac	ctcag' A	-	1	-	102	protein folding	8
BeE60H23F12	Cyclophilin D	G 'gtacg	actgac	gacag' G	-	-	1	65	protein folding	18
BeE60C1408(1)*	Cyclophilin A	G 'gtccg	actcac	tgtag' C	1	-	-	135	protein folding	
BeE60C1408(2)*	Cyclophilin A	G 'gtacg	catcac	ggaag' A	1	-	-	252	protein folding	35
BeE90D07F08(3)	Cyclophilin A	G 'gtacg	gttgac	tcggg' G	-	1	-	103	protein folding	35
BeSAS476(1)	Peptidil prolyl cis-trans isomerase (FK506)	G 'gtggg	actcac	tctag' G	2	-	-	169	protein folding	10
BeSAS476(2)*	Peptidil prolyl cis-trans isomerase (FK506)	G 'gtgcg	tctgac	tctag' G	2	-	-	78	protein folding	10
BeSAS21	Prohibitin	G 'gtggg	atcaac	tctag' T	-	2	1	70	protein folding	
BeE60C35D10(1)	Hypothetical protein	G 'gtaca	ctcaac	cgtag' A	1	-	-	62	protein modification	

BeE60C35D10(2)	Hypothetical protein	T 'gtaca	cctgac	tgtag' G	1	-	-	59	protein modification	
BeSAS143*	Ubiquitin-conjugating enzyme	G 'gtacg	actgac	cgtag' A	-	1	2	72	protein modification	
BeE60C10A07	Hypothetical protein	G 'gtgcg	actcac	cgtag' T	1	-	-	68	protein modification	9
BeSAS589(1)	26S proteasome regulatory complex subunit p37A	G 'gtacg	cctgac	accag' G	-	2	-	65	proteolysis	
BeSAS589(2)	26S proteasome regulatory complex subunit p37A	C 'gtatg	actgac	tgtag' A	-	2	-	64	proteolysis	
BeE60C19E02	ENSANG00000022234	G 'gtacg	tctcac	aacag' T	1	-	-	61	regulation of transcription, DNA-dependent	
BeSAS714	Glutathione Peroxidase	G 'gtgag	gctaac	gtcag' C	2	2	1	334	response to oxidative stress	20
BeSAS517	GTP cyclohydrolase II	G 'gtgcg	cctcac	accag' A	2	3	-	65	riboflavin biosynthesis	
BeE60C09G12*	mitochondrial phosphate carrier protein (Mir1)	A 'ctgtt	gttgac	cgtac' C	1	-	-	108	transport	
BeE60C15F01	NTF2-related export protein 2	G 'gtacg	actcac	tccag' T	1	-	-	56	transport	
BeE60H21G06*	Nuclear transport factor 2 (ntf-2)	C 'gtacg	gcttac	tgtag' C	-	-	1	57	transport	8
BeSAS199	Hypothetical protein	G 'gtacc	gctgac	tgcag' G	1	1	2	107	ubiquitin-dependent protein catabolism	
BeSAS813(1)*	Ubiquitin fusion degradation protein	C 'gtgtg	tcttac	tgtag' T	-	2	-	63	ubiquitin-dependent protein catabolism	
BeSAS813(2)	Ubiquitin fusion degradation protein	G 'gtgcg	gctgac	actag' T	-	2	-	62	ubiquitin-dependent protein catabolism	
BeSAS435	Synaptobrevin	G 'gtcgg	gctcac	tgtag' C	2	-	-	155	vesicle-mediated transport	
BeSAS548(1)	Synaptobrevin-like V-snare protein	C 'gtacg	gctgac	cgcag' C	2	-	1	65	vesicle-mediated transport	
BeSAS548(2)	Synaptobrevin-like V-snare protein	G 'gtgcg	gctcac	cacag' A	2	-	1	64	vesicle-mediated transport	
BeSAS224	Hypothetical protein	G 'gtacg	ctgaac	cacag' G	-	2	2	67		
BeSAS239*	No match	T 'gtccg	actgac	tgtag' C	-	1	2	81		
BeSAS305*	No match	G 'gtacg	acatac	tctag' T	-	-	2	63		
BeSAS377(1)	Hypothetical protein	G 'gtacg	actgac	cacag' A	1	1	-	64		
BeSAS377(2)	Hypothetical protein	G 'gtacg	actgac	tgtag' T	1	1	-	65		
BeSAS377(3)	Hypothetical protein	G 'gtacg	gctgat	tgtag' A	1	1	-	62		
BeSAS421*	Putative elicitor-responsive gene	G 'gtacg	tctcac	tctag' T	2	-	-	72		2
BeSAS428	Putative pirin-like protein	G 'gtacg	gctcac	tctag' A	2	6	-	73		27
BeSAS442*	Peroxiredoxins	G 'gtacg	gctcac	actag' G	2	-	-	120		54
BeSAS468(1)	No match	C 'gtacg	gctcac	ttcag' A	2	-	-	253		
BeSAS468(2)	No match	G 'gtgtg	tctgac	tgtag' C	2	-	-	64		
BeSAS492	No match	G 'gtgcg	ccgcat	cccag' G	1	-	1	164		
BeSAS584(1)	No match	G 'gtatg	gctcac	tctag' C	-	2	-	100		
BeSAS584(2)	No match	G 'gtacg	gctcac	cacag' A	-	2	-	65		
BeSAS585	Hypothetical protein	G 'gtgcg	gctgac	tgcag' G	2	3	3	67		
BeSAS630*	No match	G 'gtacg	gctaac	cgcag' A	1	1	3	93		
BeSAS638(1)	No match	G 'gtatg	gctcac	cacag' A	1	3	-	65		
BeSAS638(2)	No match	G 'gtgcg	gctgac	tgtag' C	1	3	-	64		
BeSAS653	Zn-dependent alcohol dehydrogenase	G 'gtacg	gctaac	tgcag' G	-	5	-	67		
BeSAS735	Microsomal glutathione S-transferase 3	G 'gtact	actgac	ggtag' C	2	1	-	77		14

BeSAS792	Membrane bound C2 domain containing protein	G 'gtgcg	gctgac	cgtag' T	2	1	-	83	6
BeSAS802	No match	C 'gtacg	gctcac	ttcag' A	2	2	3	253	2
BeSAS814	Microsomal glutathione S-transferase 3	G 'gtacg	gctaac	cctag' G	-	2	-	67	8
BeE60C10A05(1)*	No match	G 'gtacg	gctcac	cgtag' C	1	-	-	186	
BeE60C10A05(2)*	No match	G 'gtacg	acttac	tctag' T	1	-	-	63	
BeE60C10E09	RNA binding protein	G 'gtacg	gcggac	aacag' G	1	-	-	59	3
BeE60C11C05(1)*	Hypothetical protein	G 'gtaca	gttgac	tttag' C	1	-	-	72	
BeE60C11C05(2)	Hypothetical protein	G 'gtacg	tctgac	tctag' A	1	-	-	91	
BeE60C11F05	Unamed protein product	G 'gtgcg	actgac	tgtag' G	1	-	-	64	
BeE60C24F03	No match	C 'gtacg	cctcac	tgtag' C	1	-	-	67	
BeE60C34G12	Hypothetical protein	C 'gtacg	cctgac	tgtag' C	1	-	-	61	
BeE60C35G03	Hematopoietic cell-specific Lyn substrate 1	C 'gtgag	gctgac	cttag' C	1	-	-	61	
BeE60C36B06(1)	Similar to apoAI protein	T 'gtccg	gctgac	cctag' T	1	-	-	92	
BeE60C36B06(2)	Similar to apoAI protein	G 'gttcg	tctgcac	tacag' G	1	-	-	58	
BeE60C36B06(3)	Similar to apoAI protein	G 'gttcg	tctcac	tgtag' G	1	-	-	61	
BeE60H17C01*	No match	C 'gtacg	gctgat	tgtag' C	-	-	1	57	
BeE60H22D09	No match	T 'gtaca	actgac	tgtag' G	-	-	1	77	
BeE60H28F12	No match	G 'gtacg	gctcac	cgtag' C	-	-	1	187	
BeE60H28H03	No match	G 'gcgcg	gctgac	cggcg' G	-	-	1	176	
BeE60H29B06(1)	SAM-dependent methyltransferases	C 'gtacg	gctgac	gttag' G	-	-	1	61	
BeE60H29B06(2)	SAM-dependent methyltransferases	G 'gtacg	cctgac	tgtag' C	-	-	1	59	
BeE60H31A04*	No match	G 'gtgcg	tctcac	tctag' A	-	-	1	75	
BeE90D02G03*	Inositol-1(or 4)-monophosphatase 2 (IMPase 2)	G 'gtaag	gctgaa	tgtag' A	-	1	-	57	
BeE90D04E07*	HSPC177]	G 'gtgcg	cctcac	tgtag'A	-	1	-	63	
BeE90D05A10*	No match	G 'gtacg	acatac	tctag'T	-	1	-	63	
BeE90D07H07	No match	G 'gtagg	actcac	ggtag' C	-	1	-	53	2
BeE90D09D10*	Hypothetical protein	G 'gtatg	gctaac	tctag' C	-	1	-	243	
BeE90D11G08*	C2 contain domain protein	G 'gtgcg	actgac	tttag' T	-	1	-	75	12
BeE90D12F02	ENSANG00000010717	G 'gtatg	gttgac	tgtag' T	-	1	-	61	
BeE90D15D07(1)	GTP cyclohydrolase I	G 'gtacg	gctcac	cgtag' C	-	1	-	112	
BeE90D15D07(2)	GTP cyclohydrolase I	G 'gtacg	actcac	gacag' A	-	1	-	79	
BeE90D16C06*	Glutathione-S-Transferase	T 'gtacg	gctgac	tctag' G	-	1	-	72	14
BeE90D17F08	No match	G 'gtaag	attcat	accag' A	-	1	-	62	

The putative 5’ and 3’ splicing sites (SS), as well as the branch site and the intron length are shown. The biological processes in which the proteins encoded by these genes are involved are also depicted. The number of iESTs observed in each stress cDNA library (CDM, CDC or HSR) is shown. The last column indicates the number of ESTs sequenced also from stress cDNA libraries but that did not presented introns in its sequence.The numbers between parentheses indicate the number of introns observed in a same iESTs. Asterisks correspond to the ESTs that possess introns but maintain the coding frame.
