# Supplementary material for: Environmental stresses inhibit splicing in the aquatic fungus Blastocladiella emersonii
Source: BMC Microbiol. 2009 Oct 29;9:231. doi: 10.1186/1471-2180-9-231 (PMC2773782; doi:10.1186/1471-2180-9-231)
Supplement: Additional file 2 — Genes encoding spliceosome proteins in B. emersonii, annotated in GO category "mRNA processing". The table shows ESTs that participate in mRNA processing in B. emersonii. [file 1471-2180-9-231-S2.pdf]

Supplemental Table 2 – Genes encoding spliceosome proteins in *B. emersonii*, annotated in GO category "mRNA processing".

Gene ID	Annotation	Categories
BeCSAS368	mRNA capping enzyme	Cap
BeG30N18G01	mRNA cap guanine-N7 methyltransferase	Cap
BeE90D16F01	mRNA capping enzyme beta subunit	Cap
BeZSPNP17D08	mRNA decapping enzyme 1B	Cap
BeE60C32G03	LSM domain-containing protein 1	SM family
BeCSAS1385	U6 snRNA-associated Sm-like protein LSM4	SM family
BeCSAS1096	Similar to U6 snRNA-associated Sm-like protein LSM5	SM family
BeCSAS447	Small nuclear ribonucleoprotein	SM family
BeE60N15H01	Small nuclear ribonucleoprotein Sm D1	SM family
BeCSAS304	Small nuclear ribonucleoprotein Sm D2-like protein	SM family
BeCSAS1252	Small nuclear ribonucleoprotein Sm D3	SM family
BeCSAS1629	Small nuclear ribonucleoprotein Sm E	SM family
BeCSAS1658	Small nuclear ribonucleoprotein Sm F	SM family
BeE60N19C01	Small nuclear ribonucleoprotein Sm G	SM family
BeCSAS8	Sm-like protein	SM family
BeCSAS2475	SnRNP core protein SMX5d - Lsm2	SM family
BeG120N01F07	Peptidyl-prolyl isomerase Cwc27	Splicing factor
BeE30N19E11*	Pre-mRNA-splicing factor Cwc2	Splicing factor
BeE60N18G11	Pre-mRNA-splicing factor Cwc26	Splicing factor
BeCSAS558	Pre-mRNA-splicing factor Isy1	Splicing factor
BeE30N13A04	Pre-mRNA-splicing factor Prp45	Splicing factor
BeCSAS513	Pre-mRNA-splicing factor Prp46	Splicing factor
BeE90N10F12	Pre-mRNA-splicing factor Rse1	Splicing factor
BeCSAS750	Pre-mRNA-splicing factor Syf2	Splicing factor
BeE90D04E05	Probable pre-mRNA splicing factor Prp1	Splicing factor
BeCSAS813	Putative splicing factor	Splicing factor
BeE60N02E02	Splicing factor 3B subunit 2	Splicing factor
BeCSAS1462	Splicing factor 3B subunit 5	Splicing factor
BeG90N18H03	Splicing factor 3B subunit 3	Splicing factor
BeCSAS2344*	Cleavage and polyadenylation specificity factor 5	
BeE60N07A04	Cleavage stimulation factor, 3' pre-RNA	
BeE60N07D10	Cleavage stimulation factor, 50 kDa subunit	
BeE90D03C05	E3 ubiquitin protein ligase TOM1-like protein	
BeCSAS2312	SR-rich pre-mRNA splicing activator	
BeCSAS780	DUF866 domain protein	
BeE60H15D06	Similar to pre-mRNA-splicing factor Prp1	
BeCSAS566	RNA binding motif protein 8	
BeG120N03E07	Protein interacting with Hsp90 1 similar to yeast Nop17	
BeE90D13G04	Putative U5 small nuclear ribonucleoprotein 200 kDa helicase	
BeCSAS417	U4/U6 X U5 tri-snRNP complex subunit Dim1	
BeCSAS1763	WD-repeat protein Pop3	

Asterisks indicate the proteins that possess zinc-related domains.
