# Supplementary material for: Environmental stresses inhibit splicing in the aquatic fungus Blastocladiella emersonii
Source: BMC Microbiol. 2009 Oct 29;9:231. doi: 10.1186/1471-2180-9-231 (PMC2773782; doi:10.1186/1471-2180-9-231)
Supplement: Additional file 3 — S1 protection assays of hsp70 mRNA in different cadmium concentrations. The figure shows Sl protection assays of hsp70 mRNA using total RNA extracted from B. emersonii cells submitted to different cadmium concentrations. [file 1471-2180-9-231-S3.pdf]

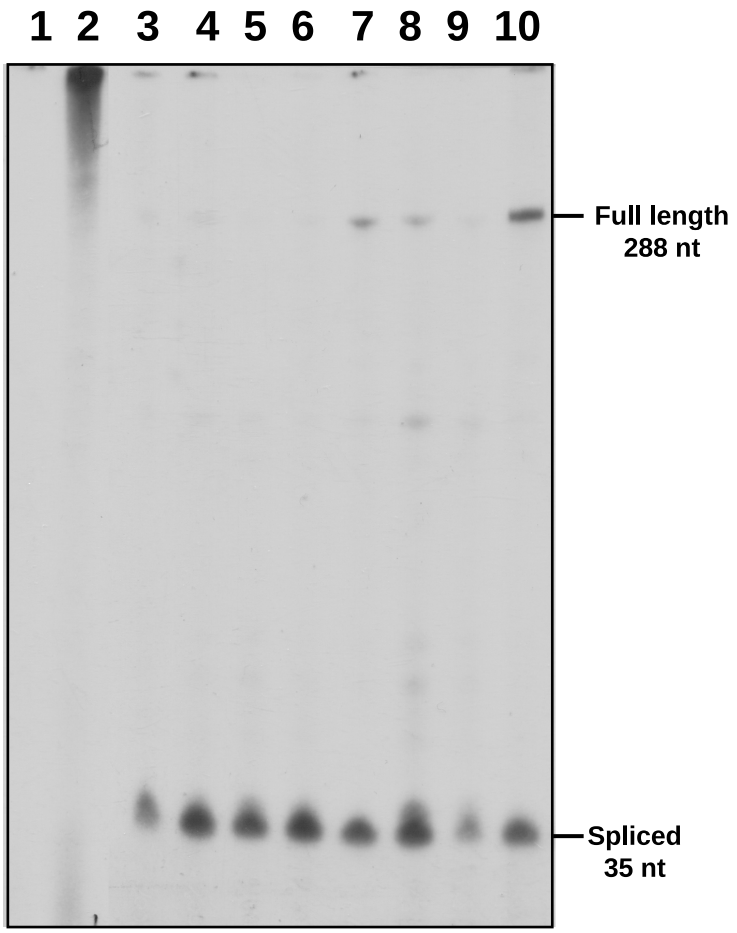

Additional file 3: S1 protection assays of *hsp70* mRNA in different cadmium concentrations. Protection assays were performed using a 5' end-labeled probe (the same used in Figure 3) and 50 µg of yeast tRNA in the presence or absence of S1 nuclease as control (lanes 1 and 2), 50 µg of total RNA isolated from 30 min and 60 min sporulating cells (lanes 3 and 4, respectively), cells submitted to 25 µM CdCl2 for 30 min and 60 min (lanes 5 and 6, respectively), cells submitted to 50 µM CdCl2 for 30 min and 60 min (lanes 7 and 8, respectively), cells submitted to 100 µM CdCl2 for 30 min and 60 min (lanes 9 and 10, respectively).
